# Preparticipation Cardiovascular Screening of Athletes: Current Controversies and Challenges for the Future

**DOI:** 10.3390/diagnostics14212445

**Published:** 2024-10-31

**Authors:** Hélder Dores, Paulo Dinis, José Miguel Viegas, António Freitas

**Affiliations:** 1 Department of Cardiology, Hospital da Luz, 1600-209 Lisbon, Portugal; 2 CHRC—Comprehensive Health Research Center, Associate Laboratory REAL (LA-REAL), 1099-085 Lisbon, Portugal; 3 NOVA Medical School, 1069-061 Lisbon, Portugal; 4 CoLab TRIALS, 7002-554 Évora, Portugal; 5 Department of Cardiology, Centro Hospitalar e Universitário de Coimbra, 3000-075 Coimbra, Portugal; paulogdinis@gmail.com; 6 Coimbra Military Health Center, Portuguese Army, 3000-075 Coimbra, Portugal; 7 Department of Cardiology, Hospital de Santa Marta, Centro Hospitalar Universitário de Lisboa Central, 1169-050 Lisbon, Portugal; miguel09@gmail.com; 8 Department of Cardiology, Hospital Professor Doutor Fernando Fonseca, 2720-276 Lisbon, Portugal; aeffreitas@gmail.com; 9 Centro de Medicina Desportiva de Lisboa, 1649-028 Lisbon, Portugal

**Keywords:** athlete, sports cardiology, sudden cardiac death, pre-participation screening, digital

## Abstract

Sports cardiology is an evolving field in cardiology, with several topics remaining controversial. Beyond the several well-known benefits of regular exercise practice, the occurrence of adverse clinical events during sports in apparently healthy individuals, especially sudden cardiac death, and the described long-term adverse cardiac adaptations associated to high volume of exercise, remain challenging. The early identification of athletes with increased risk is critical, but the most appropriate preparticipation screening protocols are also debatable and a more personalized evaluation, considering individual and sports-related characteristics, will potentially optimize this evaluation. As the risk of major clinical events during sports is not zero, independently of previous evaluation, ensuring the capacity for cardiopulmonary resuscitation, especially with availability of automated external defibrillators, in sports arenas, is crucial for its prevention and to improve outcomes. As in other areas of medicine, application of new digital technologies, including artificial intelligence, is promising and could improve in near future several aspects of sports cardiology. This paper aims to review the methodology of athletes’ preparticipation screening, emphasizing current controversies and future challenges, in order to improve early diagnosis of conditions associated with sudden cardiac death.

## 1. Introduction

Physical activity and regular exercise training are associated to multiple benefits for health, especially at cardiovascular (CV) level, improving the prevention and control of several risk factors, the quality of life and reducing the rate of cardiovascular diseases (CVD). On the other hand, physical inactivity and sedentarism are well known independent predictors for CVD, contributing for the development of several diseases and premature mortality [1].

Although the huge evidence supporting its multiple favorable benefits, in the presence of some risk factors and CVD, exercise can trigger severe clinical complications, including sudden cardiac death (SCD), affecting the broad spectrum of apparently healthy individuals, from young to veteran athletes [2]. In this setting, early identification of athletes with increased risk and with diagnosis of conditions associated with SCD in this population, is critical. Although some topics about SCD in athletes need clarification, the most common causes are well-known, especially inherited cardiac diseases in young and coronary artery disease (CAD) in older athletes (>35 years old) [3].

Preparticipation screening (PPS) allows the detection of cardiac conditions and other abnormal structural or electrical substrates that may induce fatal arrhythmias during exercise. As SCD may be the first manifestation of concealed disease in asymptomatic individuals, it is justified the mandatory and periodic evaluation of athletes [4,5]. However, the methodology for athletes’ PPS remains somewhat controversial and a more personalized assessment, considering both individual and specific sports-related characteristics, could potentially optimize this evaluation.

PPS is important to mitigate clinical complications associated with sports, but even after a detailed evaluation some conditions may not be detectable. In fact, there are variables not possible to test, mainly those associated with the environment where the sport is practiced. Subsequently, to ensure immediate capacity of cardiopulmonary resuscitation (CPR), especially with the use of automated external defibrillators (AEDs) and performed by bystanders, is a crucial measure to improve survival after sudden cardiac arrest (SCA) [3,6]. The time from collapse to defibrillation is a critical determinant of survival and focusing on the likelihood of successful resuscitation of cardiac arrests is central to prevent fatal events.

Sports cardiology is an evolving field, with several topics remaining controversial and new opportunities for research. For example, as in other areas of medicine, application of digital technologies, including artificial intelligence (AI), is promising and could be helpful to improve PPS [7]. Integrating these tools could offers several opportunities to enhance risk stratification, diagnosis, and athletes monitoring.

This paper aims to review the methodology of athletes’ PPS, emphasizing current controversies and future challenges to improve the early diagnosis of conditions associated with SCD.

## 2. Sudden Cardiac Death in Athletes

### 2.1. Epidemiology of SCD

The real incidence of SCD during participation in sports remain unknown and has varied widely across studies. Although being the leading medical cause of death in young athletes during exercise, SCD in uncommon, with a generally accepted annual incidence ranging from one to three cases per 100,000 person-years [8,9].

The differences reported are mainly derived from the heterogeneity of the methodologies used and populations analysed. The determination of SCD incidence requires an accurate identification of the number of cases per year (numerator) and the number of athletes participating in sports per year (denominator) in the population studied. Most studies included retrospective data reviewed from media reports and electronic databases, which may clearly underestimate the incidence [10]. Understanding the incidence of SCD in athletes is even limited by different definitions used for “athlete” and “SCD”.

Data from a retrospective study of the Rescu Epistry cardiac arrest database showed an incidence of SCA, during participation in competitive sports, of 0.76 cases per 100,000 athlete-years [11]. A landmark prospective study performed in the Veneto region in Italy showed an incidence of 3.6 per 100,000 athlete-years before and a significant reduction to 0.4 per 100,000 after implementation of mandatory PPS [12]. A 27-years registry in United States of America revealed an incidence of 0.6 per 100,000 athlete-years [13]. Among young athletes, a study of individuals of the Minnesota high school aged 12–19 years old showed an incidence of SCD of 0.24 per 100,000 athlete-years, while in athletes of the National Collegiate Athletic Association (17–24 years old), also in United States of America, the incidence was 1.9 per 100,000 athlete-years and in a study performed in 1,710 high schools with AEDs it was 2.2 per 100,000 athlete-years [6,8,14]. In contrast, a study performed in United Kingdom in adolescent football players showed a higher incidence of SCD (6.8 per 100,000 athlete-years) [15].

The incidence of SCD is higher in older when compared to younger athletes. This evidence was reported in a Danish study, estimating an incidence of SCD of 0.47 to 1.21 per 100,000 person-years in young competitive athletes compared to 6.64 per 100,000 person-years in older (age > 35 years) athletes [16]. In fact, athletes over 35-years old are at a 5-to-10-fold higher risk than younger, attributed to the higher prevalence of CV risk factors and CAD. Beyond age, there are other characteristics that seems to determine the incidence of SCD in athletes, such as gender, ethnicity, sporting discipline and level of competition [10].

An analysis from 3 population-based European registries showed that cardiac arrest during sport appears to be more than 10-fold higher in men compared with women, despite similar baseline characteristics and circumstances of occurrence [17]. The reasons for these differences are poorly understood, but a complex interplay between genetic, phenotypic, hormonal, and environmental mechanisms may be involved. It is important to highlight that older studies are limited by the lower proportion of competitive female athletes included, which is not the actual reality with more women participating in high-level sport.

Regarding ethnicity, some studies reported a higher risk in black athletes, exceeding that in white athletes by almost fivefold [10,18]. A 4-year prospective study based on the National Centre for Catastrophic Sports Injury Research registry in the United States of America, revealed a significant higher annual incidence rate of SCD among African American male basketball players (1:2.087 athlete-years), compared to other ethnic groups [19].

The mechanisms for which these athlete subpopulations are at disproportionately higher risk of SCD remains unclear, but for example, as African or Afro-Caribbean origin athletes are usually more involved in certain sports such as basketball, soccer, American football and long-distance running, and competitive sport has been dominated by men, these interpretations can be biased.

### 2.2. Causes of SCD in Athletes

The great majority of SCD causes in athletes are CV in origin (>80%), mainly due to inherited cardiac conditions in young, and CAD in older athletes. Among the inherited conditions, cardiomyopathies remain important causes, but cases occurring in structurally normal hearts, indicating sudden arrhythmic death syndrome (SADS) in which a primary arrhythmia is the most likely underlying etiology, have had increasing preponderance in recent studies [20].

The FIFA SCD Registry [3] proved this trend, with the leading cause in players >35 years being CAD (76%) and in players ≤35 years sudden unexplained death (22%). However, among these younger players the leading cause varied by region, being mainly cardiomyopathy in South America (42%), coronary artery anomaly in North America (33%) and sudden unexplained death in Europe (26%), which can reflect different PPS methodologies, emergency support, diseases’ prevalence and genetic background.

Regarding cardiomyopathies, hypertrophic cardiomyopathy has been reported as the most common, followed by arrhythmogenic right ventricular cardiomyopathy, especially in the Italian registry, and dilated cardiomyopathy [8,12,13,15]. Finocchiaro G [21] showed that SADS was the most common cause of SCD (63%) in young individuals, followed by myocardial diseases (22%), especially arrhythmogenic cardiomyopathy, hypertrophic cardiomyopathy, idiopathic left ventricular hypertrophy, and myocarditis. In the Peterson study [19], the most frequent causes in young athletes were hypertrophic cardiomyopathy (20.6%), idiopathic left ventricular hypertrophy (13.4%), coronary artery anomalies (12.0%) and autopsy-negative sudden unexplained death (9.6%). A recent published large specialist pathology registry revealed SADS as the most common cause of SCD (53%) followed by cardiomyopathies (22%) [20].

It is important to stress the importance of autopsy in SCD cases, as death certification alone can be inaccurate, and the autopsy imaging is not yet reliable enough in detecting specific cardiac diseases. However, such as was shown in the FIFA SCD Registry, the rate of autopsies in footballers suffering SCD is very low, with a specific cause being documented in 211 cases (34% of all cases), of which only 124 were identified by the autopsy [3]. Since SCD cases in younger athletes are largely due to genetic conditions, it is essential to improve risk stratification and develop preventative strategies for their family members, who are also at risk. Establishing molecular autopsy in evaluation of SCD victims could be a very important step.

**Figure 1:**
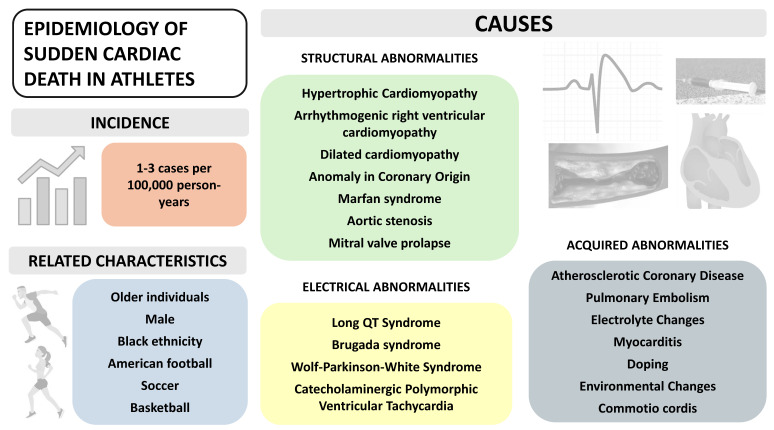
Most relevant messages about the epidemiology of SCD in athletes.

Myocarditis is also a relevant cause of SCD in athletes, accounting for 2% to 9% of cases and fatal arrhythmias may be the first clinical manifestation of the disease [22]. Coronary artery anomalies have been reported as the second most frequent cause of SCD among young athletes in the United States of America, accounting for 17% of deaths, while in a recent study from the United Kingdom were responsible for 9% of deaths among competitive adolescent athletes [13,21,22]. Environmental factors such as heat stroke, commotio cordis and performance-enhancing drugs are also linked with SCD in athletes and should be considered [22]. Figure 1 presents the most relevant messages about SCD in athletes.

### 2.3. Controversies and Challenges for the Future

The incidence of SCD remains wide due to methodological inconsistencies in the previous published studies.

Understanding the characteristics associated with higher risk for SCD and its variance according to sports disciplines are needed.

Developing mandatory prospective registries with systematic report of all SCD cases in athletes is crucial.

It is important to standardize the postmortem evaluation of SCD victims, with application of new tools and technologies such as molecular evaluation.

## 3. Preparticipation Cardiac Screening of Young Athletes

### 3.1. Clinical Evaluation

In young athletes PPS should be focus on a complete personal and family medical history, physical examination and 12-lead electrocardiography (ECG) [4,23]. The presence of symptoms such as exertional chest pain, palpitations, dizziness or syncope should be seen as red flags. The education of the athletes to not train in the presence of fever or before a complete recovery from an acute illness is an important recommendation. Since most of SCD causes in young athletes are genetically determined, knowing family clinical background is essential. In this setting, history of cardiomyopathy, channelopathy or sudden unexplained death in the family should lead to further investigation [24]. In the physical examination, excluding abnormal heart murmur (any systolic or diastolic grade >II/VI), elevated blood pressure, delayed femoral pulse, and irregular heart rhythm, as well as being aware of musculoskeletal and ocular features suggestive of Marfan syndrome, are central aspects [23,24].

### 3.2. The Role of Electrocardiogram

Optimal PPS is still a matter of debate. In Europe, several scientific societies including the European Society of Cardiology (ESC), advocate the use of ECG as an initial part of PPS, complementary to clinical history and physical examination [4]. On the other hand, both the American Heart Association (AHA) and the American College of Cardiology (ACC) are against mandatory ECG screening for sports participation, but recognise that this exam can be important in high-risk groups, including in some high level sports, or in community based programs with sufficient resources and when ECG interpretation is performed by trained sports cardiologists [25,26].

The key evidence supporting the use of ECG comes from the Corrado et al [27]. prospective study, where systematic ECG screening reduced dramatically SCD rate from 3.6% to 0.4%. Compared to clinical history and physical examination alone, ECG increases the sensitivity and specificity [25]. However, AHA and ACC criticize this model due to the high economic impact, logistics burden and number of false positive cases, which lead to additional assessments with substantial costs and unnecessary athlete’s disqualifications, supporting a methodology based on clinical history and physical examination.

Differentiating between physiological and pathological conditions is essential to correctly identify those athletes who are at higher risk. The interpretation and appropriate application of ECG criteria in athletes is a critical step to ensure high quality and reduce the false positives results. In the last two decades, the criteria for ECG interpretation in athletes have been improved and disseminated. Currently, the International Criteria, published in 2017 by a panel of experts in Cardiology and Sports Medicine, with superior accuracy and significant reduction of false positives cases, are recommended [28].

Despite this improvement, ECG interpretation in athletes requires training and some patterns are often misinterpreted by clinicians with less experience. Common findings that are misjudged include: left ventricular hypertrophy, nonpathological T wave inversion (TWI), isolated right bundle branch block, J waves and nonpathological rhythm variants [29]. Some of these changes are the result of physiologic and benign electrical cardiac remodeling. Although International Criteria are currently the most accurate in identifying athletes at risk, there is scope to improvement. Therefore, it is important to consider the athlete’s geographic origin, race, gender, age and type of sports. Recent evidence suggests that other ECG findings may have clinical significance such as low QRS voltage, QRS fragmentation, ST-segment depressions and premature ventricular contraction (PVC) morphology. Table 1 presents some aspects that need further clarification regarding the influence of athletes’ or exercise-related characteristics on electrical remodeling and the relevance of some specific ECG findings in athletes.

Among other aspects that need clarification to optimize PPS in athletes, are the definition of the most appropriate time for the first screening and the frequency of these evaluations [30,31]. In Italy, all individuals who participate in organized sports need annual medical evaluation, and the initiation is determined by the different sports associations. In previous data from the Veneto region, PPS was done in athletes aged 12 to 35 years old [29], and International Criteria were designed for application in athletes aged 12–35 years old [30]. On the other hand, Sarto et al [31]. evaluated 22.234 children-athletes aged 7–18 years old and repeated the PPS annually, concluding that the PPS is beneficial in all ages, increases the diagnostic yield in the group of children aged 12–18 years old, with a superior cost per diagnosis in the group aged 7–11 years old (EUR 140 041 vs. EUR 69 957), and that repeating evaluations are essential, since the probability of identifying an athlete at risk is similar comparing the first with the follow-up evaluations. In this study, more diagnosis were significantly established on repeated evaluations than at the initial PPS (64% vs. 36%).

### 3.3. Inclusion of Echocardiogram

Transthoracic echocardiogram (TTE) can improve the accuracy of PPS, as it can detect some pathologies associated with SCD, such as anomalous origin of the coronary arteries, aortic bicuspid valve and mitral valve prolapse, that would not be identified in a regular PPS [32]. However, adding TTE to a PPS comes with a cost. It brings more complexity to the PPS, with substantial financial burden to the society, requires heavier logistics in the initial PPS and follow up, more comprehensive additional assessments, and is operator dependent requiring learning training period, skills and echocardiography experience [33].

To minimize costs and save time, some authors and scientific societies advocate the use of focused TTE protocols [34,35]. The British Society of Echocardiography published a document for the use of TTE in screening young athletes aged 14 to 35 years old. This screening is not mandatory as a first line investigation, and the specific sport organization has the authority to decide in which athletes TTE is required. Regarding the first TTE evaluation, most authors suggest performing the exam in adolescence (12–14 years old) [34]. Although one TTE during adolescence will exclude congenital heart diseases, it may miss the presence of cardiomyopathies that may only develop phenotypic expression later. This point highlights the need for future re-evaluation of these athletes, however, the timing to repeat the TTE is not yet well established (1 to 5 years interval).

### 3.4. Controversies and Challenges for the Future

Understanding the potential influence of specific sports and exercise-related characteristics on cardiac physiological adaptations, especial at electrical level, is needed to optimize PPS.

Additional research is essential to understand the clinical significance of some ECG patterns (e.g., low QRS voltage, QRS fragmentation, PVC morphology).

Definition of the most appropriate age for the first PPS, its frequency and whether it should vary according to the type of sport should be clarified.

Better understanding on the role of TTE in athletes PPS, the protocol to use, and timing for re-evaluation remains unknown.

## 4. Screening of Veteran Athletes

A veteran athlete is usually defined as an individual older than 35 years involved in regular physical exercise with recreational or competitive purposes. The 35-year age cutoff is a simplifying binary definition for a heterogeneous population regarding the prevalence of CVD, risk profile, and risk related to sports participation. Other designations like adult, master, middle-aged, senior, and older athletes are also commonly used.

Emerging evidence shows that chronic exposure to large amounts of exercise may predispose individuals to some adverse findings like arrhythmias, myocardial fibrosis, and increased coronary atherosclerotic burden [36,37,38,39]. On the other hand, the growing popularity of some ultra-endurance running and extreme sporting events attracts many individuals, often with low fitness levels and with CV risk factors, who wish to challenge their own physical limits. Although CAD is the most prevalent cause of major CV events in veteran athletes, causes such as arrhythmias, aortic disease, and silent unknown CVD, should not be ignored [36]. Furthermore, myocardial scar is more common in veteran athletes, especially in males, and its suspicion must be raised, particularly in athletes with a significant burden of PVC [40].

### 4.1. Cardiovascular Risk Stratification of Veteran Athletes

**Figure 2:**
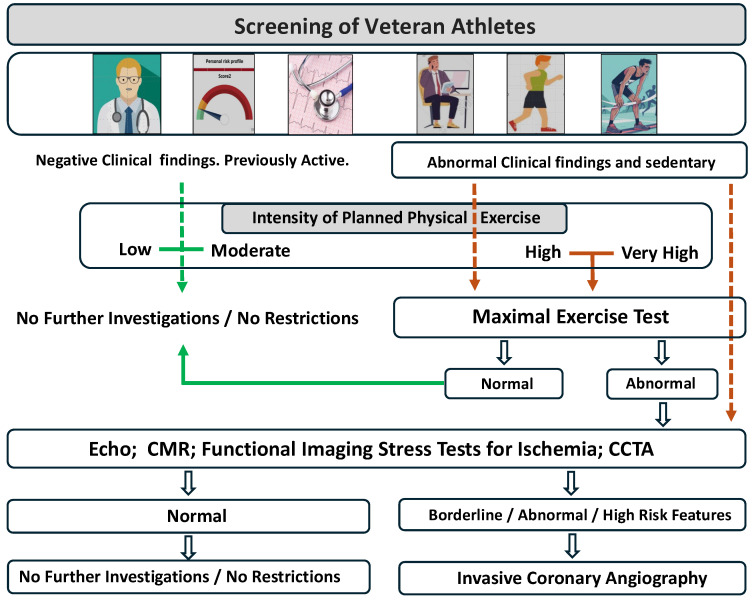
Proposed algorithm for screening veteran athlete (adapted and modified from ESC [4]).

PPS methodology recommended for veteran athletes is depicted in Figure 2. CV risk stratification should be a stepwise process where clinical evaluation is in the first line, including the investigation of symptoms, family history of premature CAD or SCD, CV risk stratification, physical examination, and a resting ECG. In addition to the traditional CV risk factors, the usual level of physical activity and intensity of the planned exercise, especially if high intensity, are important factors to be considered in the PPS, as suggested by the ESC and American College of Sports Medicine (ACSM) recommendations [4,41,42].

In general, asymptomatic individuals without CV risk factors, previously active, who intend to start mild to moderate-intensity exercise, can be cleared without further investigation. The main message is “be active.” Screening should not be a barrier for a mild to moderate exercise. The presence of symptoms, positive family history, high-risk Systematic Coronary Risk Evaluation (SCORE) system or Framingham Risk Score (FRS), middle-aged and older athletes, or those previously sedentary, require further additional investigation. Veteran athletes performing high-intensity exercise, particularly long-distance races or extreme sports, may also need a more complete screening protocol.

### 4.2. The Role of Exercise Testing

For many years, exercise testing (ET) has been used as part of the screening of asymptomatic individuals with increased CV risk factors who intend to start regular exercise training [41]. The wide availability and relative low cost, along with being one of the most physiological functional tests, makes ET very useful in the evaluation of middle-aged and older individuals with CV risk factors and sedentary individuals who intend to initiate vigorous or high-intensity exercise. CV screening in adult and senior athletes must target the higher prevalence of CAD. However, the diagnostic accuracy of ET in asymptomatic individuals without CV risk factors is debatable. Although ET may be sensitive in identifying obstructive CAD, it may not detect non-significant stenosis due to vulnerable plaques that can rupture and lead to acute coronary events during exercise [43].

In fact, the use of ET for routine screening of CAD in asymptomatic adults has limited evidence, with a low predictive value and a high number of false positive cases, especially in women. However, it is important to emphasize that ET, in addition to ischemic assessment, may provide a wide range of information like blood pressure response, exercise-induced arrhythmias, and symptoms triggered by maximal exercise, which are also important in CV risk assessment of veteran athletes. Some authors recommend a widespread or even systematic use of ET in veteran athletes given its capacity for additional diagnosis of other unsuspected CVDs [44]. Consistent with the 2020 ESC recommendations [4], ET should be reserved for symptomatic athletes or those deemed at high risk of CAD, based on SCORE system or equivalent FRS.

### 4.3. Further Exams and Advanced Imaging Evaluation

In the presence of abnormal clinical findings, high-risk clinical score and positive or borderline ET, it is important to exclude structural heart disease and rule out CAD. Computed Coronary Tomography Angiography (CCTA) offers the best way to evaluate the coronary tree, the presence of plaques, their morphology, extension, degree of obstruction, and global coronary atherosclerotic burden [38,39,40].

In the presence of obstructive lesions, further evaluation is required for detecting inducible myocardial ischemia, using advanced imaging modalities, preferably with exercise rather than pharmacological stress. Advanced cardiac imaging tests are usually used as a third-line evaluation, when there are abnormal findings in the previous tests, but are not recommended as a routine screening. Despite this fact, CCTA has been presented as a promising tool for screening asymptomatic veteran athletes, due to its proven value over traditional clinical risk factors in predicting atherosclerotic heart disease among individuals with low to moderate CV risk. However, despite the limitations of traditional PPS methodology in raising suspicion of CAD, there are several arguments against the systematic screening of asymptomatic low-risk athletes with CTCA [44,45]. The financial costs of screening a low-risk population, the potential harm of contrast reactions and radiation exposure are worrying aspects to consider. According to the most recent recommendations, the systematic use of CCTA in asymptomatic veteran athletes with a normal ET is not recommended [4].

In the first line the clinical evaluation, CV Risk Score, previously activity level an intensity of planned exercise (see text). Maximal ET is recommended for individuals with abnormal clinical evaluation, previously sedentary or those who intend to start a high or very high intensity exercise program. When there are abnormal findings on first-line evaluation, it is essential to exclude structural heart diseases, particularly valvular or “myopathic” heart disease, as well as coronary atherosclerotic disease or induced ischemia (CCTA: Computed Coronary Tomography Angiography; CMR: Cardiovascular Magnetic Resonance).

### 4.4. Controversies and Challenges for the Future

There are still many “mysteries” about cardiac adaptation to long-term endurance sport and its prognostic impact, especially in female athletes.

The traditional PPS protocols are limited in identifying athletes at risk and the evidence-based recommendations to screen veteran athletes is scarce.

Robust longitudinal studies comparing outcomes and cost-effectiveness of the different PPS protocols are still missing and should be developed.

The importance of promoting an active lifestyle and regular sports participation, with a prudent balance maximizing benefits and minimizing risks, should not be underestimated.

## 5. Importance of Cardiopulmonary Resuscitation

SCA during sports is a traumatic event with profound emotional and psychological impacts on the athlete’s family, team, and broader community. Despite advances in CV screening, the prevention of sports-related SCA (SrSCA) remains a significant challenge [46]. While PPS aims to identify individuals at risk, it is not infallible, and no screening program can provide complete protection against SrSCA [47]. Consequently, the limitations of PPS highlight the critical need for robust secondary prevention strategies. These strategies should emphasize the importance of bystander CPR and the prompt use of AEDs to enable early defibrillation. The prompt recognition of SCA is a critical first step in initiating a rapid and effective emergency response. Coaches, sports medicine professionals, and other anticipated first responders must maintain a high index of suspicion for SCA in any athlete who experiences sudden collapse and becomes unresponsive.

The implementation of an effective emergency action plan (EAP) is paramount in improving survival outcomes following SrSCA, by ensuring a coordinated and efficient response to such emergencies [48]. For these response programs to be successful, they must feature an organized and rehearsed protocol, an efficient communication system to activate emergency medical services, and personnel trained and equipped to perform early CPR and defibrillation. AEDs should be strategically placed in schools and sports facilities to ensure that the time from collapse to the first shock is less than three minutes, with optimal accessibility to minimize any delay [49]. The main limitation of EAPs is the cost associated with training and AED maintenance. Hence, demonstrating its impact on survival is essential to emphasize the need for greater efforts in the development of EAPs during both competitive events and recreational activities.

### 5.1. Impact in Survival After Sudden Cardiac Arrest

The most common mechanism of SrSCA is adrenergic-triggered ventricular fibrillation, often resulting from an underlying clinically silent CV condition [50]. The time from collapse to defibrillation is a critical factor influencing survival after SCA, with survival rates decreasing by 7% to 10% for every minute of delay [15]. Ischemic brain injury following cardiac arrest is a significant predictor of both short-term mortality and long-term neurological outcomes in out-of-hospital cardiac arrest survivors [51].

Numerous studies have demonstrated that immediate CPR and early defibrillation using on-site AEDs are pivotal in reducing mortality and minimizing post-anoxic brain injury. For instance, a study using a Luxembourg nationwide database revealed that the survival rate for patients who received bystander CPR during cardiac arrest was approximately 50%, while those who did not receive CPR all succumbed to the event [51]. Another recent study by Karam et al [52]. examined trends in SrSCA incidence, management, and survival in the Greater Paris area from 2005 to 2018. While the incidence of SrSCA remained stable, suggesting a need for improved screening strategies, there was a consistent increase in bystander CPR initiation and AED use over time. As the chain of survival was strengthened, these improvements resulted in a threefold increase in survival rates, highlighting the critical role of bystander CPR and AED access in improving outcomes. Further, a meta-analysis by Michelland et al [53]. demonstrated that bystander presence, bystander CPR, and bystander AED use were associated with two-, three-, and five-fold increases in survival without neurological damage, respectively. Bystander AED use showed the most significant impact on survival, reinforcing the need for proper EAPs, witness intervention, and widespread availability of AEDs in sports facilities. Notably, these survival benefits were observed in both competitive and recreational athletes.

Sports associations advocate for EAPs that ensure team coaches, trainers, and medical staff are trained in CPR. However, while athletes are often closest to an individual experiencing SrSCA, current guidelines do not mandate CPR training for athletes themselves. Given that every minute of delay decreases survival, immediate intervention by those nearest to the collapsed athlete, often teammates, could be crucial. A retrospective review by Chukumerije et al [54]. found that in 91% of SrSCA cases, CPR was performed by non-athlete personnel, with only 9% of cases involving athlete-initiated CPR. In a related survey, only 50% of athletes reported knowing what SCA is, and just 8% expressed concern about SCA during play. These findings underscore the low awareness and limited concern about SrSCA among athletes, contributing to the low rate of lay-rescuer athlete CPR. Given that trained medical personnel are less likely to be present during amateur events than competitive ones, it is vital that fellow athletes initiate CPR when no staff are available. Even in settings where medical staff are present, immediate CPR by athletes could significantly improve survival outcomes.

The presence of AEDs in sports centers and during competitions should be mandated; however, sports are often practiced outside formal facilities, especially at the amateur level, where the risk of SCA is no less significant. Indeed, most sports-related sudden deaths occur in amateur settings, which are frequently outdoors. In Europe, 66% of cardiac arrests are witnessed, but fewer than half of these cases involve bystander intervention. If all witnesses were equipped to act, up to 100,000 lives could be saved annually [55]. Thus, there is an urgent need for broader availability of AEDs and increased training in basic life support, aiming for a state of “widespread cardioprotection” [47]. To achieve this, it is essential to expand the pool of potential rescuers who can recognize cardiac arrest and initiate CPR. Research from the United States has shown that CPR training rates are higher in states with mandatory CPR education, particularly in schools [56]. Encouraging CPR training from a young age may play a crucial role in preparing more individuals to intervene effectively in cases of SCA.

Management of survivals after SCA is challenging. Beyond the clinical aspects, with the need for implantable cardioverter defibrillator (ICD) for secondary prevention, the decision regarding sports eligibility in athletes is controversial. A shared decision-making process should be considered for the participation in highly intensive or competitive sports, taking into consideration the effect of exercise on the underlying substrate, its role as a trigger for appropriate or inappropriate ICD shocks and the psychological impact of shocks. In this setting, it is also important to highlight that ICD implantation is not recommended as a substitute for disease-related recommendations when these mandate sports restrictions [4].

### 5.2. Controversies and Challenges for the Future

SrSCA is often witnessed, providing an opportunity for rapid intervention. However, many cases still experience delayed and suboptimal resuscitation, even when medical teams equipped with AEDs are present on the sidelines.

The recent successful recoveries of some athletes are emblematic of the remarkable advances in SrSCA treatment in recent years and should serve as a strategic opportunity to further promote awareness programs aimed at training bystanders in basic lifesaving skills (Table 2).

Countries should prioritize the development of comprehensive first aid training policies and ensure AED availability in sports settings. Although some nations already mandate AEDs in sports facilities and during competitions, this requirement should be implemented globally.

Concerted efforts are needed, including legislation mandating public AED access, along with tax incentives to support deployment. Extending these initiatives beyond sports settings is essential to improve outcomes for all instances of SCA, regardless of location. Additionally, integrating basic life support training into schools, at maturity age, or upon receiving a driver’s license could significantly expand public education, ultimately increasing the number of potential life-saving responders.

## 6. The Role of Digital Technology

### 6.1. Where Are We?

In recent decades, there has been a significant surge in the development and utilization of digital technologies within the field of sports cardiology [57]. These innovations offer promising new tools for the early detection of potential cardiac conditions in athletes and assist clinicians in distinguishing between physiological and pathological adaptations. Furthermore, these technologies enable continuous monitoring beyond initial PPS, thereby mitigating the risk of SCD by identifying early indicators of cardiac distress [7].

Advanced ECG and imaging software have revolutionized the precision of data collection during cardiac assessments [58]. These tools are instrumental in detecting asymptomatic heart abnormalities that may pose a risk during sports. Moreover, the increasing interest in consumer wearable devices (CWDs), such as activity trackers and heart rate monitors, is reshaping the landscape of sports medicine [59]. Photoplethysmography (PPG), the primary technology employed by CWDs for health metric measurements, is particularly prevalent in wrist- and finger-worn devices [60]. The evolution of sensor technology has advanced to the point where ECG recordings via surface electrodes are now feasible. This capability is crucial for the identification of major arrhythmias [61]. By providing a comprehensive range of cardiopulmonary metrics directly to consumers, wearable technology holds significant potential for large-scale remote monitoring and early detection of cardiac irregularities, thereby enhancing preventive care. The integration of data from these devices can also guide training intensity, allowing for designed training regimens and personalized exercise prescriptions tailored to athletes with cardiac conditions [7]. Additionally, digital platforms have improved communication between athletes, coaches, and medical teams, ensuring that relevant health data is shared promptly and efficiently. Mobile applications and cloud-based systems enable remote monitoring, which is particularly advantageous for continuous cardiac assessment, especially in high-risk athletes [7].

### 6.2. Artificial Intelligence in Sports Cardiology

AI has experienced remarkable growth over the past decade, with its applications increasingly explored across various sectors of the healthcare system. Sports cardiology is no exception, as AI’s potential is beginning to be recognized through a growing number of studies specifically focused on athletes [62]. Although still in its early stages, AI application in this field shows great promise.

AI methodologies have demonstrated a significant ability to process vast amounts of medical data, identify patterns, make predictions, and support physicians in critical decision-making processes [63]. The integration of AI technologies has the potential to revolutionize sports cardiology by improving the early detection and prevention of cardiac anomalies, refining risk assessment, and enhancing treatment planning and monitoring for athletes [62]. This innovation offers a promising pathway to transform traditional screening practices into more advanced, precise, and predictive healthcare interventions. Table 3 outlines emerging AI tools and applications in sports cardiology, particularly highlighting their potential to enhance PPS and reduce the risk of SCD in athletes.

### 6.3. Controversies and Challenges for the Future

AI algorithms capable of identifying subtle signs of disease could enable more accurate and timely PPS for athletes across all levels of sport, and may enhance understanding of athletes’ cardiac dynamics, paving the way for more effective and personalized cardiac health strategies.

Integration of AI in sports cardiology is challenging, including considerable costs and potential threats such as technological dependency and the risk of data misuse.

New CWDs with a wide range of reported metrics often enter the market before undergoing thorough scientific testing to fully establish their efficacy and safety, leaving both clinicians and patients to navigate complex data without the support of definitive validation. Athletes may experience unnecessary distress and over-investigation upon discovering abnormalities on their CWDs.

Ethically, issues such as informed consent, transparency, algorithmic fairness, bias, and data privacy must be scrutinized to ensure responsible use. Legally, concerns regarding safety, liability, data protection, cybersecurity, and intellectual property rights must be addressed to protect both patients and healthcare providers.

## 7. Conclusions

Athletes’ PPS is a central topic in sports cardiology, but with several controversies and challenges remaining to clarify, in order to improve early identification of individuals at risk. The reported incidence of SCD is inconsistent, while the definition of the characteristics associated with higher risk for SCD and its variance according to sports disciplines is not established. In this setting, developing mandatory prospective registries with systematic reporting of all SCD cases in athletes is crucial. Furthermore, establishing appropriate PPS protocols for athletes’ evaluation, especially veteran athletes, is a priority in this field. Robust longitudinal studies comparing outcomes and cost-effectiveness of the different protocols should be developed. Additionally, the evidence of long-term adverse effects and cardiac maladaptations among endurance athletes is concerning and should be investigated with more detail. Beyond the optimization of PPS, development of appropriate EAP and policies, ensuring comprehensive training on CPR and AED availability in sports, should be globally implemented to improve survival outcomes following SrSCA. The integration of digital technologies and AI into sports cardiology may potentially enhance athletes’ evaluation, but as this field is evolving, robust research and regulatory frameworks will be critical in unlocking the full potential of these transformative technologies.

## Figures and Tables

**Table 1:** Aspects that need further clarification regarding the influence of athletes’ or exercise-related characteristics on electrical remodeling and the relevance of specific ECG findings.

ATHLETE’S SPECIFIC CHARACTERISTICS	
**Geographic origin and race**	Black athlete repolarization pattern (J-point elevation and TWI in V1–V4) is considered a normal finding; however, it shows heterogeneous patterns of benignity in different geographic regions or African population.
**Age and gender**	More research and normative ECG data on age and gender is needed. Some ECG parameters are dynamic and change with age (QT interval, QRS voltage). TWI in V1–V3 is more prevalent in female athletes (benign pattern?).
**SPORTS SPECIFIC CHARACTERISTICS**	
**Type of sport/Modality**	Cardiac remodeling, both structural and electrical, is dependent on the type of sports. Endurance athletes have predominantly eccentric remodeling and strength athletes a concentric type.
**Intensity**	Elite athletes show more marked physiological cardiac adaptations. Future research should be focus on normative ECG data according to exercise-related characteristics.
**SPECIFIC ECG FINDINGS**	
**Low QRS voltage**	Causes of SCD such as arrhythmogenic cardiomyopathy, myocarditis, infiltrative cardiac diseases and nonischemic left ventricle scar may demonstrate low QRS voltage. In athletes this is infrequent.
**QRS fragmentation**	In lead V1 it may represent a sign of right ventricle remodeling. More research is needed to understand its clinical value.
**Premature Ventricular Contractions**	Beyond the PVC burden, the morphology and relationship with exercise matters. PVC from concerning origins (e.g., Left bundle branch block with superior or intermediate axis) warrant an indepth investigation.
**ST-segment depression morphology**	Upsloping ST-segment depression can have a different value (benign), than a horizontal or downsloping ST-depression morphology. Research is needed.

ECG: Electrocardiogram; PVC: Premature Ventricular Contractions; SCD: Sudden Cardiac Death.

**Table 2:** Essential Strategies for Managing Sports-Related Sudden Cardiac Arrest.

Key Measure	Description
**Improved Screening Strategies**	Enhance CV PPS to better identify at-risk athletes.
**Emergency Action Plans**	Implement well-rehearsed EAPs in sports venues, ensuring fast and efficient response.
**Bystander Training**	Provide widespread CPR and AED training to coaches, medical staff, and spectators to ensure rapid response.
**Athlete CPR Training**	Encourage CPR training for athletes to improve immediate intervention.
**Mandatory AED Availability**	Enforce the presence of AEDs in both competitive and recreational sports settings.
**Legislative and Tax Incentives**	Promote AED accessibility laws and provide tax incentives for deployment and training.
**Education in Schools**	Incorporate basic life support and AED training into school curricula to increase awareness.

AED: automated external defibrillator; CPR: cardiopulmonary resuscitation; CV: cardiovascular; EAP: emergency action plan; PPS: pre-participation screening.

**Table 3:** The emerging role of AI in sports cardiology.

**History and Physical Examination**	**Anamnestic Data Collection**: AI-powered chatbots facilitate the collection of medical history by streamlining the process and identifying critical details that might otherwise be missed during conventional clinical visits [63].
	**AI-Enhanced Stethoscopes**: Digital stethoscopes equipped with heart murmur interpretation algorithms improve the detection of structural heart murmurs in athletes [64].
**Electrocardiography**	**ECG Interpretation**: AI models provide enhanced precision in interpreting ECG results, offering improved detection of CV conditions linked to SCD and the ability to predict clinical outcomes [65].
	**Exercise Testing**: Advanced AI algorithms enhance the diagnostic accuracy of exercise stress tests and continuous ECG monitoring, particularly in identifying coronary artery disease and heart rhythm disorders [66].
**Imaging**	**Echocardiography**: AI-based tools analyse echocardiographic patterns, aiding clinicians in differentiating physiological athletic heart adaptations from pathological conditions [67].
	**Advanced Imaging**: AI-driven analysis in cardiac magnetic resonance imaging, coronary computed tomography, and nuclear cardiology assists in diagnosing conditions within the “grey zone” of athlete’s heart [7].
**Genetic Testing**	**Genomic Data Analysis**: AI models applied to large genomic datasets help identify genetic markers and disease subtypes, enabling more accurate risk stratification and supporting personalized treatment strategies [68].
**Wearable Technology**	**Continuous Monitoring**: AI integration with wearable devices extends their capabilities, enabling continuous cardiac monitoring and enhanced risk assessment in athletes [60].

AI: artificial intelligence; CV: cardiovascular; ECG: eletrocardiograma; SCD: sudden cardiac death.
